# Are blood tests being performed for new inpatient admissions to a psychiatric hospital as recommended by RCPsych guidelines?

**DOI:** 10.1192/bjo.2021.295

**Published:** 2021-06-18

**Authors:** Isabella Piper

**Affiliations:** Leverndale Psychiatric Hospital

## Abstract

**Aims:**

Severe mental illness (SMI) has a significant impact on a person's physical health and mortality. There is a 10–25-year life expectancy reduction in patients with SMI. The majority of deaths are due to physical health conditions. The Royal College of Psychiatry (RCPsych) sets out a standard that new inpatient admissions to Mental Health Services should have routine blood tests performed within 24 hours of admission, unless they have had a recent blood test. The aim of this audit was to review whether blood tests were performed either in the 48 hours preceding admission or the 48 hours after admission to Leverndale Hospital.

**Method:**

Clinical records were reviewed for new inpatient admissions to two general adult wards over a four-month period.

**Result:**

79 patients were admitted (M = 39, F = 40, Age: 18–62 years old). 70/79 (89%) had blood tests performed within the 48-hour timeframe. 5/79 (6%) had a blood test performed after 48 hours of their admission. 4/79 (5%) did not have a blood test. The blood tests performed varied. 51/75 (68%) patients had at least one abnormal blood test. The yield of abnormal blood results ranged from 2% for thyroid function tests to 35% for a full blood count.

**Conclusion:**

This audit has established that the majority of patients had blood tests performed within the 48-hour timeframe. This could be improved by setting up an electronic reminder to prompt the clinician to perform a blood test at 24 hours as per RCPsych guidance if one had not yet been done. The blood tests performed varied. RCPsych guidance does not specify which blood tests should be done. A further scope for this audit could be to review the clinical significance of abnormal blood results to develop a standard set of blood tests for admission.

